# A185 A GUT MICROBIOME TRANSITION: THE JOURNEY FROM INDIA TO CANADA

**DOI:** 10.1093/jcag/gwad061.185

**Published:** 2024-02-14

**Authors:** L D D'Aloisio, N Haskey, N Abulizi, J Barnett, V Shetty, U Bhaumik, M Ballal, S Ghosh, D Gibson

**Affiliations:** Biology, The University of British Columbia Okanagan, Kelowna, BC, Canada; University of Calgary, Calgary, AB, Canada; Biology, The University of British Columbia Okanagan, Kelowna, BC, Canada; Biology, The University of British Columbia Okanagan, Kelowna, BC, Canada; Kasturba Medical College Manipal, Manipal, Karnataka, India; University of Maryland School of Medicine, Baltimore, MD; Kasturba Medical College Manipal, Manipal, Karnataka, India; Biology, The University of British Columbia Okanagan, Kelowna, BC, Canada; Biology, The University of British Columbia Okanagan, Kelowna, BC, Canada

## Abstract

**Background:**

Young Indian immigrants and Indo-Canadians face a significantly higher risk of inflammatory bowel disease (IBD) in westernized countries. While the etiology of IBD remains unclear, a gut microbiome that is no longer symbiotic with its host is a key player. However, Indians are underrepresented in microbiome research, therefore we cannot accurately assess the role of their gut microbiome in IBD. To understand why Indians are at a greater risk for IBD in Canada, we must first characterize their gut microbiome.

**Aims:**

This study explores how immigration to Canada impacts the gut microbiome of Indian populations, potentially elevating their risk to IBD.

**Methods:**

Stool samples from healthy volunteers (ages 17-53) were collected from Indians in India, Indo-Immigrants, Indo-Canadians, Euro-Canadians and Euro-Immigrants. DNA was extracted for 16S sequencing on the Illumina MiSeq platform and shotgun sequencing on the Illumina NovaSeq platform. Dietary data was processed in ESHA.

**Results:**

Weighted Unifrac revealed distinct clusters of Indian and Indo-Immigrant samples from the westernized cohorts. The most pronounced difference was between Indian and Euro-Canadians with a pseudo-F value of 49.18 (q = 0.00125). Indian versus Indo-Immigrants resulted in a pseudo-F value of 7.707 (q = 0.00125), and Indian versus Indo-Canadian had a pseudo-F value of 15.95 (q = 0.00125). BugBase estimated a significantly higher stress tolerance in the Indian gut, with Proteobacteria driving this phenotypic prediction. Compared to Euro-Canadians, a significantly higher pathogenic potential was predicted in Indians (*p*_*FDR*_*=* 1.04e-11) and Indo-Immigrants (*p*_*FDR*_*=* 1.41e*-*04), driven mainly from Proteobacteria and Bacteroidetes. Multiple comparisons with LDA revealed *Prevotella* was over 5 times more abundant in Indians residing in India. The gut microbiome in Indians were also enriched with taxa associated with a plant-based diet. Dietary data showed that 59% of Indians do not consume meat, and they also had the lowest consumption (12% caloric intake) of group 4 ultra-processed food (UPF), whereas the highest consumption of UPF was in Indo-Canadians (61% caloric intake) (*p*_*bonf*_ ampersand:003C 0.0001). A frequent trend was observed in Indians living in Canada, which was a reduction in key nutrients including soluble fiber, zinc, iron, and folate, some of which are commonly found in plant-based foods.

**Conclusions:**

Overall findings reveal that a loss of *Prevotella* abundance was observed in Indo-Immigrants and Indo-Canadians, which was associated with a reduction in key nutrients that are common to a plant-based diet, indicating a transition away from their traditional diet. Their dietary changes may be a driver to the displacement of *Prevotella* and other taxa commonly found in the Indian gut microbiome.

**Funding Agencies:**

NSERC, Killam

